# Targeted cellular metabolism for cancer chemotherapy with recombinant arginine-degrading enzymes

**DOI:** 10.18632/oncotarget.135

**Published:** 2010-08-04

**Authors:** Macus Tien Kuo, Niramol Savaraj, Lynn G. Feun

**Affiliations:** ^1^Department of Molecular Pathology, The University of Texas M. D. Anderson Cancer Center, Houston, TX, USA; ^2^Sylverster Comprehensive Cancer Center, University of Miami Miller School of Medicine, Miami, FL, USA; ^3^Division of Hematology and Oncology, Miami Veterans Affairs Healthcare System, Miami, FL, USA

**Keywords:** arginine, auxotrophy, ADI-PEG20, arginase, targeted therapy, drug resistance

## Abstract

It has been shown that a subset of human cancers, notably, melanoma and hepatocellular carcinoma (HCC) are auxotrophic for arginine (Arg), because they do not express argininosuccinate synthetase (ASS), the rate-limiting enzyme for the biosynthesis of arginine from citrulline. These ASS-negative cancer cells require Arg from extracellular sources for survival. When they are exposed to recombinant Arg-degrading enzymes, e.g. arginine deiminase (ADI) or arginase, they die because of Arg starvation; whereas normal cells which express ASS are able to survive. A pegylated ADI (ADI-PEG20) has been developed for clinical trials for advanced melanoma and HCC; and favorable results have been obtained. ADI-PEG20 treatment induces autophagy in auxotrophic cancer cells leading to cell death. Clinical studies in melanoma patients show that re-expression of ASS is associated with ADI-PEG20 resistance. ADI-PEG20 treatment down-regulates the expression of HIF-1α but up-regulates c-Myc in culture melanoma cells. Induction of ASS by ADI-PEG20 involves positive regulators c-Myc and Sp4 and negative regulator HIF1α. Since both HIF-1α and c-Myc play important roles in cancer cell energy metabolism, together these results suggest that targeted cancer cell metabolism through modulation of HIF-1α and c-Myc expression may improve the efficacy of ADI-PEG20 in treating Arg auxotrophic tumors.

## Altered tumor metabolism as target for cancer chemotherapy

Dysregulation of cellular metabolism is a hallmark of many human malignancies. The metabolic differences between normal cells and tumor cells have provided opportunities for developing novel approaches for the diagnosis and treatment of cancer. The “Warburg effect” which describes the preferentially metabolizing glucose via anaerobic glycolysis instead of oxidative phosphorylation found in many cancers, led to the development of 2-fluoro-2-deoxyglucose positron emission tomography [^18^FDG-PET] as a valuable oncology imaging tool [1]. Enhanced proliferative capacities found in tumor cells associated with aberrations of many signal transduction pathways resulting from genetic or epigenetic changes in oncogenes and/or tumor suppressor genes has led to the development of many targeted therapeutics for treating many types of malignancies. At the present time, most of our understanding about the dysregulated cancer cell metabolism remains at the “gross” physiological stages. As technological development advances, we may eventually be able to differentiate the metabolic differences between cancerous and normal cells at the single-tumor level, ultimately leading to the development of personalized cancer medicine. In this review, we focus on the recent development of targeted therapy of a subset of human malignancies with altered Arg metabolism.

## Arginine-deficiency and auxotrophy in cancer cells

**Figure 1:**
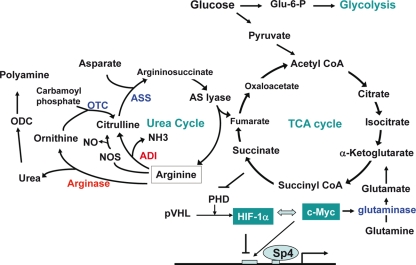
Metabolic pathways relevant to Arg deprivation strategy using ADI-PEG20 Shown in the center are the coupled TCA and urea cycles. In the urea cycle, arginine is metabolized by arginase to citrulline and urea. Arginine can be metabolized into citrulline and nitric oxide (NO) by nitroxide synthetase (NOS). Citrulline is converted into argininosuccinate (AS) by argininosuccinate synthetase (ASS). AS is recycled back to arginine by argininosuccinate lyase. In most melanoma cells, AS is not active, therefore, arginine must come from an external source. Treating melanoma cells with arginine deiminase (ADI), which converts arginine to citrulline and ammonia, results in arginine deprivation, leading to cell death in melanoma cells. We found that ASS expression can be induced in some melanoma cells involving the interplay of c-Myc/HIF-1α/Sp4. The relationships of this metabolic wiring and the function of HIF-1α and c-Myc are also indicated.

Arg is an intermediate metabolite in the urea cycle. *De novo* biosynthesis of Arg requires two sequential enzymatic steps: argininosuccinate synthetase (ASS) which catalyzes the synthesis of argininosuccinate from L-citrulline and aspartic acid, and argininosuccinate lyase (ASL) which converts argininosuccinate into L-Arg and fumaric acid (Fig. 1); of which, ASS is the rate-limiting enzyme. Fumarate is an important metabolite of tricarboxylic acid (TCA) cycle, linking Arg metabolism to glucose-generated energy metabolism. Moreover, Arg is involved in many other important cellular metabolic pathways, including the biosyntheses of polyamine, creatine and nitric oxide, nucleotides, proline and glutamate biosyntheses [2-4].

In normal cells, ASS is a ubiquitous enzyme but its level of expression differs among different cell types and can be regulated by many extracellular factors. Expression of hepatic ASS can be transcriptionally regulated by cyclic AMP [5] and endothelial ASS expression is regulated by cytokines such as IL-1, TNF-α, and TGF-β1 and glutamate [6,7]. Levels of ASS vary markedly in a wide spectrum of tumor tissues as compared with their corresponding normal counterparts. Elevated levels of ASS expression have been found in cancers of the ovary, stomach, and colon. By contrast, reduced or undetectable levels of ASS have been found in the majority of melanoma, hepatocellular carcinoma (HCC), mesotheliomas, renal cell carcinoma, and prostate cancers [8-11]. The mechanisms that control ASS expression in these tumor types remain elusive. The ASS-negative tumors are unable to survive if the systemic Arg supply is depleted. Therefore, they are auxotrophic for Arg.

## Targeted therapy of Ass-negative cancers with arginine-degrading enzymes

Two recombinant proteins that degrade Arg have been under development for treating Arg auxotropic tumors: ADI and arginase. ADI catabolizes the conversion of arginine to citrulline and ammonia; and arginase degrades arginine to ornithine which is then converted to citrulline by ornithine carbamoyl transferase (OCT), an additional step for arginine-to-citrulline conversion (Fig. 1).

ADI is a bacterial enzyme secreted from *Mycoplasma* spp. into cultures. Sugimura et al [12] were the first to identify that ADI from *M. arginini* is a lymphoblastogenesis inhibitory factor. Miyazaki et al [13] reported that ADI purified from *Mycoplasma*-derived culture medium potently inhibits the growth of human tumor cells. Takaku et al [14] described the *in vivo* antitumor activity of ADI in melanoma animal models [15,16]. However, because bacterial ADI has short half-life (~ 4 hr) in the circulation and is highly immunogenic, Holtsberg et al [17] formulated a pegADI with poly(ethylene glycol) of molecular mass of 20,000 Daltons for clinical use, termed ADI-PEG20. Phase I-II clinical trial in patients with advanced or metastatic melanoma showed antitumor activities, including partial and complete responses [18]. A 35% response rate has been seen with minimal toxicity [19]. Importantly, the response and stable diseases were only seen in patients with tumors that do not express ASS (L.F & NS, unpublished clinical data). Compared with the poor response rates (<20%) of malignant melanoma to current standard treatment options [20], ADI-PEG20 represents a novel approach for the targeted therapy of advanced melanoma. A phase I/IIa trial (n = 19) [21] and a recent phase II trial [22] of ADI-PEG20 for human HCC showed the effectiveness of HCC treatment. In these studies, ADI-PEG20 treatment was well tolerated and effectively reduced serum Arg levels without adverse effects. However, antigenicity against ADI-PEG20 eventually arose about 50 days after the treatment [22].

The antigenicity associated with ADI-PEG20 treatment has motivated the development of recombinant human arginase (rhArg1) for targeting Arg auxotrophic tumors. While arginase is about 1000 times less potent as compared with ADI under physiologic condition [13], rhArg1 has also been under development for treating HCC [23]. Optimal activity for native arginase is at pH ~9.5, making it less active at physiological pH [24]. hArg1 contains two coordinated Mn^2+^ ions at its active site. Replacing Mn^2+^ with Co^2+^ coordination in rhArg1 reduces its pH optimum to pH 7.4 and enhances its cytotoxic effects (~ 10-fold) to hepatoma cells in culture [25]. Thus, this bioengineered arginase warrants further clinical study.

## Mechanisms of cell killing in targeted therapy of L-Arg auxotrophic cancer cells by arginine-depleting enzymes

While amino acid deprivation is known to induce nutritional starvation, how the depletion of a single amino acid such as Arg leads to cell death is not well-understood. ADI-PEG20- or arginase-induced cell death in cultured cells usually takes 3 to 6 days. However, early events such as induction of autophagy occurs can be detected 90 min. after the start of treatment [26,27] and can last for over 72 hr [28]. Autophagy is a lysosomal degradation pathway which involves vesicular sequestration of proteins or organelles into autophagasomes which then fuse with lysosomes and become degraded [29,30]. Autophagy occurs upon nutrition stress as a means to protect cells from apoptosis by re-cycle amino acid from protein degradation. In this regard, global metabolic stress could be detected in prostate cancer animal model 4 hr after ADI-PEG20 treatment as measured by ^18^FDG-PET [26]. Furthermore, activation of nutrient/energy-sensing pathway, which involves upregulation of AMPK/mTOR/S6K, is accompanied with the induction of autophagy in ASS-negative cells treated with ADI-PEG20. Several studies have shown that prolonged treatment with ADI-PEG20 in cultured cells induces apoptosis [27,31,32]. The exposure time that leads to apoptosis and the extent of apoptosis induced by ADI-PEG20 treatments are different among melanoma cell lines and may be related to the antiapoptotic machinery in the cells [27,33].

## Mechanisms of resistance to ADI-PEG20 mediated by the induction of argninosuccinate synthetase expression

One important finding in our phase I/II melanoma clinical trial with ADI-PEG20 was that patients who were initially ASS negative could become ASS positive upon relapse, suggesting that induction of ASS expression in tumors is likely to be a mechanism of ADI resistance. To investigate the induction mechanism of ASS expression by ADI-PEG20, we used three melanoma cell lines A2058, SK-MEL-2, and A375. These cell lines express undetectable levels of ASS. When grown in ADI-PEG20-containing medium or Arg-free medium, induction of ASS expression was seen in A2058 and SK-MEL-2 cells but not in A375 cells. Induction of ASS expression is associated with ADI-PEG20 resistance, which can be reversed when ASS expression is knocked down with siRNA, suggesting that ASS expression is correlated with ADI-PEG20 sensitivity [34]. Further investigation revealed that the positive transcriptional regulator c-Myc and the negative transcriptional regulator HIF-1α interacting with the E-box element located at the promoter of the *ASS* gene (34) controls the expression of *ASS*.

Before ADI-PEG20 treatment, HIF-1α binds to the E-box and silences *ASS* expression. Upon ADI treatment, HIF-1α level is rapidly downregulated (t½ ~ 45 min.) and c-Myc level is upregulated. c-Myc replaces HIF-1α binding to the promoter of *ASS* and transactivates the expression of *ASS*. No upregulation of c-Myc was found in ADI-PEG20-treated A375 cells and no switch between HIF-1α and c-Myc binding was found in A375 cells upon ADI-PEG20 treatment [34]. ADI-PEG20-resistant variants were found in A2058 cells and SK-MEL-2 cells but not in A375 cells. Thus, the inducibility of altered HIF-1α and c-Myc expression may be a predictive signal for the ADI-PEG20 sensitivity of cells as well as the ultimate development of drug resistance.

The observation that Arg deprivation induces up-regulation of c-Myc but down-regulation of HIF-1α in some melanoma cells but not in others is intriguing. c-Myc plays a central role in a transcriptional network that regulates cell growth, differentiation, apoptosis, and metabolic signaling [35,36], and it has been demonstrated that c-Myc depletion inhibits cell proliferation in many types of human tumor cells [37]. In melanoma cells, c-Myc depletion induces senescence reminiscent of normal melanocytes [38]. Likewise, HIF-1α is also involved in the regulation of tumor growth, angiogenesis, and invasion [39]. Given the recent discoveries that c-Myc and HIF-1α are involved in the regulation of cancer energy metabolism [40,41] (Fig. 1), our finding that c-Myc and HIF-1α have opposite responses to ADI-PEG20 treatment has important implications beyond the induction of ASS and ADI-PEG20 sensitivity, it underscores the global effects on cancer cell energy metabolism as well.

## CONCLUSIONS AND FUTURE DIRECTIONS

Targeted therapy of cancers auxotrophic for Arg in humans using Arg-degrading enzymes, e.g. ADI-PEG20, has been investigated at many cancer centers and promising results have been obtained. These types of cancers generally are difficult to treat with conventional chemotherapeutics. One important aspect of ADI-PEG20 treatment is that patients are well-tolerated because of low adverse side effects. This finding provides an opportunity to improve therapeutic efficacy by combining ADI-PEG20 with other antitumor agents. Previous studies have demonstrated that ADI-PEG20 treatment induces nutritional stress and activation of autophagy response, leading to cell death. Future development may combine the use of autophagy-targeting drugs to enhance the therapeutic effect of ADI-PEG20. Moreover, we have found that ADI-PEG20 treatment induces ASS re-expression which is associated with the emergence of ADI-PEG20 resistance. We have demonstrated the roles of c-Myc and HIF-1α in the regulation of *ASS* expression, providing a rationale for developing c-Myc-[42] and HIF-1α-targeting drugs [43] to combat the evolution of drug resistance. Finally, it remains important to elucidate the underlying mechanisms by which Arg deprivation regulates c-Myc and HIF-1α that may have global effect on cancer metabolism in the Arg auxotrophic cancer cells. This research may eventually lead to the development of effective therapeutics for targeting cancer cell metabolism in general and Arg auxotrophic cancer in particular.

## References

[1] Kelloff GJ, Hoffman JM, Johnson B, Scher HI, Siegel BA, Cheng E, Cheson BD, O’Shaughnessy J, Guyton KZ, Mankoff DA, Shankar L, Larson SM, Sigman CC, Schilsky RL, Sullivan DC (2005). Progress and promise of FDG-PET imaging for cancer patient management and oncologic drug development. Clin Cancer Res.

[2] Husson A, Brasse-Lagnel C, Fairand A, Renouf S, Lavoinne A (2003). Argininosuccinate synthetase from the urea cycle to the citrulline-NO cycle. Eur J Biochem.

[3] Lind DS (2004). Arginine and cancer. J Nutr.

[4] Morris SM (2006). Arginine: beyond protein. Am J Clin Nutr.

[5] Guei TR, Liu MC, Yang CP, Su TS (2008). Identification of a liver-specific cAMP response element in the human argininosuccinate synthetase gene. Biochem Biophys Res Commun.

[6] Brasse-Lagnel C, Lavoinne A, Fairand A, Vavasseur K, Husson A (2005). IL-1beta stimulates argininosuccinate synthetase gene expression through NF-kappaB in Caco-2 cells. Biochimie.

[7] Brasse-Lagnel C, Fairand A, Lavoinne A, Husson A (2003). Glutamine stimulates argininosuccinate synthetase gene expression through cytosolic O-glycosylation of Sp1 in Caco-2 cells. J Biol Chem.

[8] Dillon BJ, Prieto VG, Curley SA, Ensor CM, Holtsberg FW, Bomalaski JS, Clark MA (2004). Incidence and distribution of argininosuccinate synthetase deficiency in human cancers: a method for identifying cancers sensitive to arginine deprivation. Cancer.

[9] Bowles TL, Kim R, Galante J, Parsons CM, Virudachalam S, Kunng HJ, Bold RJ (2008). Pancreatic cancer cell lines deficient in argininosuccinate synthetase are sensitive to arginine deprivation by arginine deiminase. Int J Cancer.

[10] Scott L, Lamb J, Smith S, Wheatley DN (2000). Single amino acid (arginine) deprivation: rapid and selective death of cultured transformed and malignant cells1. Br J Cancer.

[11] Sugimura K, Ohno T, Kusuyama T, Azuma I (1992). High sensitivity of human melanoma cell lines to the growth inhibitory activity of mycoplasmal arginine deiminase in vitro1. Melanoma Res.

[12] Sugimura K, Fukuda S, Wada Y, Taniai M, Suzuki M, Kimura T, Ohno T, Yamamoto K, Zurma I (1990). Identification and purification of arginine deiminase that originated from Mycoplasma arginini. Infect Immun.

[13] Miyazaki K, Takaku H, Umeda M, Fujita T, Huang WD, Kimura T, Yamashita J, Horio T (1990). Potent growth inhibition of human tumor cells in culture by arginine deiminase purified from a culture medium of a Mycoplasma-infected cell line. Cancer Res.

[14] Takaku H, Takase M, Abe S, Hayashi H, Miyazaki K (1992). In vivo anti-tumor activity of arginine deiminase purified from Mycoplasma arginini. Int J Cancer.

[15] Takaku H, Takase M, Abe S, Hayashi H, Miyazaki K (1992). In vivo anti-tumor activity of arginine deiminase purified from Mycoplasma arginini. Int J Cancer.

[16] Takaku H, Matsumoto M, Misawa S, Miyazaki K (1995). Anti-tumor activity of arginine deiminase from Mycoplasma argini and its growth-inhibitory mechanism. Jpn J Cancer Res.

[17] Holtsberg FW, Ensor CM, Steiner MR, Bomalaski JS, Clark MA (2002). Poly(ethylene glycol) (PEG) conjugated arginine deiminase: effects of PEG formulations on its pharmacological properties. J Control Release.

[18] Feun L, Savaraj N (2006). Pegylated arginine deiminase: a novel anticancer enzyme agent. Expert Opin Investig Drugs.

[19] Feun L, Savaraj N (2006). Pegylated arginine deiminase: a novel anticancer enzyme agent. Expert Opin Investig Drugs.

[20] Gray-Schopfer V, Wellbrock C, Marais R (2007). Melanoma biology and new targeted therapy. Nature.

[21] Izzo F, Marra P, Beneduce G, Castello G, De Rosa V, Cremona F, Ensor CM, Holtsberg FW, Bomalaski JS, Clark MA, Ng C, Curley SA (2004). Pegylated arginine deiminase treatment of patients with unresectable hepatocellular carcinoma: results from phase I/II studies. J Clin Oncol.

[22] Glazer ES, Piccirillo M, Albino V, Di Giacomo R, Palaia R, Mastro AA, Beneduce G, Castello G, De Rosa V, Petrillo A, Ascierto PA, Curley SA, Izzo F (2010). Phase II study of pegylated arginine deiminase for nonresectable and metastatic hepatocellular carcinoma. J Clin Oncol.

[23] Tsui SM, Lam WM, Lam TL, Chong HC, So PK, Kwok SY, Arnold S, Cheng PN, Wheatley DN, Lo WH, Leung YC (2009). Pegylated derivatives of recombinant human arginase (rhArg1) for sustained in vivo activity in cancer therapy: preparation, and analysis of their pharmacodynamics in vivo and in vitro and action upon hepatocellular carcinoma cell (HCC). Cancer Cell Int.

[24] Kuhn NJ, Talbot J, Ward S (1991). pH-sensitive control of arginase by Mn(II) ions at submicromolar concentrations. Arch Biochem Biophys.

[25] Stone EM, Glazer ES, Chantranupong L, Cherukuri P, Breece RM, Tierney DL, Curley SA, Iverson BL, Georgiou G (2010). Replacing Mn(2+) with Co(2+) in human arginase enhances cytotoxicity toward l-arginine auxotrophic cancer cell lines. ACS Chem Biol.

[26] Kim RH, Coates JM, Bowles TL, McNerney GP, Sutcliffe J, Jung JU, Gandour-Edwards R, Chuang FY, Bold RJ, Kung HJ (2009). Arginine deiminase as a novel therapy for prostate cancer induces autophagy and caspase-independent apoptosis. Cancer Res.

[27] Savaraj N, Wu C, Kuo M, You M, Wangpaichitr M, Robles C, Spector S, Feun L (2007). The relationship of arginine deprivation, argininosuccinate synthetase and cell death in melanoma. Drug Target Insights.

[28] Savaraj N, You M, Wu C, Wangpaichitr M, Kuo MT, Feun LG Arginine deprivation, autophagy, apoptosis (AAA) for the treatment of melanoma. Curr Mol Med.

[29] Maiuri MC, Zalckvar E, Kimchi A, Kroemer G (2007). Self-eating and self-killing: crosstalk between autophagy and apoptosis. Nat Rev Mol Cell Biol.

[30] Cao Y, Klionsky DJ (2007). Physiological functions of Atg6/Beclin 1: a unique autophagy-related protein. Cell Res.

[31] Feun L, Wu C, Kuo M, Wangpaichitr M, Spector S, Savaraj N (2007). Arginine deprivation as a targeted therapy for cancer. Current Phama Design.

[32] Gong H, Zolzer F, von Recklinghausen G, Havers W, Schweigerer L (2000). Arginine deiminase inhibits proliferation of human leukemia cells more potently than asparaginase by inducing cell cycle arrest and apoptosis. Leukemia.

[33] You M, Savaraj N, Wangpaichitr M, Wu C, Kuo MT, Varona-Santos J, Nguyen DM, Feun L The combination of ADI-PEG20 and TRAIL effectively increases cell death in melanoma cell lines. Biochem Biophys Res Commun.

[34] Tsai WB, Aiba I, Lee SY, Feun L, Savaraj N, Kuo MT (2009). Resistance to arginine deiminase treatment in melanoma cells is associated with induced argininosuccinate synthetase expression involving c-Myc/HIF-1alpha/Sp4. Mol Cancer Ther.

[35] Grandori C, Cowley SM, James LP, Eisenman RN (2000). The Myc/Max/Mad network and the transcriptional control of cell behavior. Annu Rev Cell Dev Biol.

[36] Zhang H, Gao P, Fukuda R, Kumar G, Krishnamachary B, Zeller KI, Dang CV, Semenza GL (2007). HIF-1 inhibits mitochondrial biogenesis and cellular respiration in VHL-deficient renal cell carcinoma by repression of C-MYC activity. Cancer Cell.

[37] Wang H, Mannava S, Grachtchouk V, Zhuang D, Soengas MS, Gudkov AV, Prochownik EV, Nikiforov MA (2008). c-Myc depletion inhibits proliferation of human tumor cells at various stages of the cell cycle. Oncogene.

[38] Zhuang D, Mannava S, Grachtchouk V, Tang WH, Patil S, Wawrzyniak JA, Berman AE, Giordano TJ, Prochownik EV, Soengas MS, Nikiforov MA (2008). C-MYC overexpression is required for continuous suppression of oncogene-induced senescence in melanoma cells. Oncogene.

[39] Semenza GL Defining the role of hypoxia-inducible factor 1 in cancer biology and therapeutics. Oncogene.

[40] Vander Heiden MG, Cantley LC, Thompson CB (2009). Understanding the Warburg effect: the metabolic requirements of cell proliferation. Science.

[41] Dang CV, Kim JW, Gao P, Yustein J (2008). The interplay between MYC and HIF in cancer. Nat Rev Cancer.

[42] Mo H, Henriksson M (2006). Identification of small molecules that induce apoptosis in a Myc-dependent manner and inhibit Myc-driven transformation. Proc Natl Acad Sci U S A.

[43] Semenza GL (2009). HIF-1 inhibitors for cancer therapy: from gene expression to drug discovery. Curr Pharm Des.

